# Prevention of invasive ventilation (PRiVENT)—a prospective, mixed-methods interventional, multicentre study with a parallel comparison group: study protocol

**DOI:** 10.1186/s12913-023-09283-0

**Published:** 2023-03-30

**Authors:** Julia D. Michels, Jan Meis, Noemi Sturm, Florian Bornitz, Selina von Schumann, Aline Weis, Benjamin Neetz, Martina Bentner, Johanna Forstner, Nicola Litke, Michel Wensing, Stella Erdmann, Thomas Grobe, Timm Frerk, Axel Kempa, Claus Neurohr, Armin Schneider, Michael Müller, Felix J. F. Herth, Joachim Szecsenyi, Franziska C. Trudzinski, the PRiVENT-Study Group, Franziska Christina Trudzinski, Gabriele Iberl, Beatrice Müller, Elena Biehler, Thomas Fleischhhauer, Gerhard Fuchs, Markus Qreini, Janina Schubert-Haack, Anja Klingenberg, Alex Kempa, Biljana Joves, Andreas Rheinhold, Alessandro Ghiani, Nina Lutz, Swenja Walcher, Konstantinos Tsitouras, Joanna Paderewska, Selina Briese, Christoph Andritschky, Patrick Gehrig, Joachim Sugg, Susanne Hirschmann, Simone Britsch, Christa Straub, Claude Jabbour, Michael Hahn, Jörg Krebs, Peter-Tobias Graf, Petra Denzer, Mascha O. Fiedler, Miriane Bomeken, Sebastian Stier, Tom Terboven, Uta Merle, Jens Regula, Jens Müller, Ute Oltmanns, Marcus Hennersdorf, Neslihan Satir, Mathias Borst, Brigitte Mayer, Wolfgang Reikow, Markus Kredel, Konstantin Frey, Holger Wolff, Florian Seidlitz, Stefanie Bientzle, Boris Nohé, Sebastian Allgäuer, Alexej Schöpp, Christoph Schlegel, Imke Hübner, Andrezj Kuzniar, Helene Häberle, Reimer Riessen, Benjamin Schempf, Ingo Rebenschütz, Andreas Straub, Marc Kollum, Markus Winter, Paul Hartveg, Andreas Junginger, Helmut Beck, Mathias Vogel

**Affiliations:** 1 Department of Pneumology and Critical Care, Heidelberg, Translational Lung Research Center Heidelberg (TLRC-H), Member of the German Center for Lung Research (DZL), Thoraxklinik Heidelberg gGmbH, Heidelberg, Germany; 2 Department of Pneumology and Critical Care Medicine, Thoraxklinik University of Heidelberg, Röntgenstrasse 1, Heidelberg, D-69126 Germany; 3 Institute of Medical Biometry, Heidelberg University, Heidelberg, Germany; 4 Department of General Practice and Health Services Research, University Hospital Heidelberg, Heidelberg, Germany; 5 Asklepios Hospital Barmbek, Pneumology and Internal Intensive Care Medicine, Hamburg, Germany; 6 aQua Institute for Applied Quality Improvement and Research in Health Care, Göttingen, Germany; 7 Department of Pneumology and Critical Care, SLK-Klinik Löwenstein, Löwenstein, Germany; 8 Department of Pneumology and Respiratory Medicine, Robert-Bosch-Krankenhaus Klinik Schillerhöhe, Gerlingen, Germany; 9 Department of Anaesthesia and Intensive Care Medicine Waldburg-Zeil Kliniken, Wangen Im Allgäu, Germany

**Keywords:** Invasive mechanical ventilation, Weaning, Weaning failure, Respiratory failure, Medical education and training

## Abstract

**Background:**

Invasive mechanical ventilation (IMV) is a standard therapy for intensive care patients with respiratory failure. With increasing population age and multimorbidity, the number of patients who cannot be weaned from IMV increases, resulting in impaired quality of life and high costs. In addition, human resources are tied up in the care of these patients.

**Methods:**

The PRiVENT intervention is a prospective, mixed-methods interventional, multicentre study with a parallel comparison group selected from insurance claims data of the health insurer Allgemeine Ortskrankenkasse Baden-Württemberg (AOK-BW) conducted in Baden-Württemberg, Germany, over 24 months. Four weaning centres supervise 40 intensive care units (ICUs), that are responsible for patient recruitment. The primary outcome, successful weaning from IMV, will be evaluated using a mixed logistic regression model. Secondary outcomes will be evaluated using mixed regression models.

**Discussion:**

The overall objective of the PRiVENT project is the evaluation of strategies to prevent long-term IMV. Additional objectives aim to improve weaning expertise in and cooperation with the adjacent Intensive Care Units.

**Trial registration:**

This study is registered at ClinicalTrials.gov (NCT05260853).

**Supplementary Information:**

The online version contains supplementary material available at 10.1186/s12913-023-09283-0.

## Background

Medical advances, increased life expectancy [1, 2], and higher morbidity have led to an increase in the number of people who survive prolonged intensive care [3] but still require invasive mechanical ventilation (IMV) thereafter [4]. Whether this is also true for ventilated COVID-19 patients is unclear.

Weaning from IMV is a complex and often lengthy process with the goal of cessation of IMV. According to the Budapest Conference of 2005 [5] and the current German guideline on prolonged weaning [6], different subgroups of weaning can be distinguished ranging from discharge without any further ventilatory support to discharge with invasive ventilation. The perceived quality of life of patients with out-of-hospital continuation of IMV is reduced, especially for elderly patients with chronic lung disease, regardless of whether they are cared for at home or in nursing facilities [7, 8]. Comprehensive care and specialist care in the out-patient setting are also problematic for those invasively ventilated, due to complicated in-hospital visitation [9, 10]. In addition to being dependent on IMV and requiring assistance, long-term IMV also places a burden on the affected family members [11]. Caregivers are left to their own devices, exposed to high levels of stress and as a result are at increased risk of mental illness [12]. Furthermore, health care costs increase due to the need for intensive care [13]. Patients with long-term IMV account for about 10% of intensive care cases in Germany, but tie up about 50% of available resources [14]. Without registries it is difficult to estimate the number of people receiving care outside of hospitals after unsuccessful prolonged weaning. Currently, it must be assumed that about 15,000 people are affected nationwide, with an annual increase of about 10% [11]. There are a number of specialised hospitals that offer units to wean patients of IMV following acute medical care [15]. These so-called weaning centres can be certified by the German Society for Pneumology and Respiratory Medicine (DGP) if they meet the required criteria [16]. Unfortunately, to date these specialised centres treat only a small portion of patients undergoing prolonged weaning. In Germany, up to 85% of patients discharged home with IMV did not have access to a certified weaning centre [13, 17, 18]. Several studies worldwide have shown that 60–80% of patients discharged from non-specialised ICUs with a “non-weanable” classification could still be weaned from IMV after admission to a specialised weaning centre [17–22]. However, this rate decreases when multiple comorbidities are present [18, 23, 24] and the longer the patient was ventilated before transfer to a specialised centre [25]. In addition, in-patient and post-discharge costs increase with the number of ventilator days [26]. Furthermore, the 1-year survival rate of patients with long-term IMV in an out-patient setting is estimated at 62% according to Mifsud et al. [21].

Studies, as PRiVENT, to evaluate interventions in order to reduce long-term IMV are desperately needed from a medical and health economical point of view.

## Methods/design

### Study objectives

The PRiVENT project assesses the effects of an early intervention by weaning specialists on the number of patients requiring out-of-hospital long-term IMV after hospitalisation on an ICU.

Additional objectives are:

- To reduce the number of ICU patients, who are classified as weaning failures and are not re-admitted to specialised centres
- To identify patients requiring specialised weaning therapy in non-specialised ICUs at an early stage to optimise their treatment success
- Improving the quality of weaning therapy in non-specialised ICUs and, if necessary, transferring high-risk patients in a timely manner
- Raising public awareness of weaning therapy
- Estimating the costs associated with invasive long-term ventilation.
- Determining the (additional) costs and savings associated with the PRiVENT intervention
- Assessment of patients’ quality of life during and after invasive long-term ventilation and estimation of the costs and savings related to the impact of the intervention on patients’ quality of life

The project duration is 4 years starting in July 2020. In the first 12 months, the intervention elements were developed, the study protocol was refined, and the participating ICUs were recruited. Furthermore, a small monocentric pilot study was conducted in the preparatory phase to test the individual study elements. The project aims to improve the nursing and medical expertise in the participating ICUs by establishing a weaning consultation with the specialised weaning centres.

In light of the COVID-19 pandemic, patients requiring IMV due to COVID-19 pneumonia should also benefit from the interdisciplinary treatment and care network of the PRiVENT study. Because the prognostic model was developed based on historical health care claims data before the COVID-19 outbreak, prediction of these patients’ risk of long-term IMV was not possible. However, recent studies [27, 28] indicate that patients with acute COVID-19 disease often develop acute kidney injury or cardiovascular events which increases their risk of prolonged weaning. Benito et al. [28] conclude that further studies are needed to determine prognostic factors that might predict weaning success in patients with COVID-19. Altona et al. [29] also suggest that the younger generation, including those without concomitant disease, should be studied more closely. Therefore, patients with COVID-19 pneumonia are generally assigned to the high-risk group and thus participate in the PRiVENT intervention.

In parallel, the PRiVENT process and health economic evaluation will be performed. The process evaluation is described in a separate manuscript in preparation.

### Study design

**Fig. 1:**
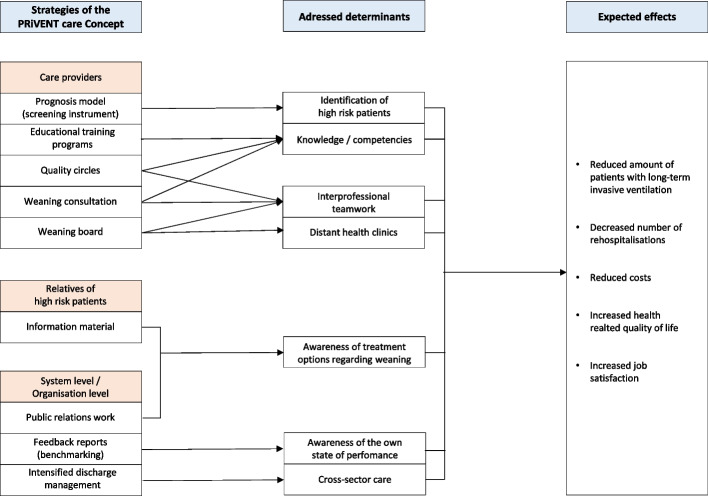
Logic Model of the PRiVENT intervention

The PRiVENT study is a prospective, interventional, unblinded, non-randomised multicentre study in an in-patient setting with a parallel comparison group. The study will be conducted with 4 weaning centres and 40 ICUs in Baden-Württemberg, Germany. See Supplement for list of weaning centres and associated ICUs. After 6 months of follow-up, analysis will be compared with a group generated from health claims data of the AOK-BW. The intervention will occur at multiple levels (Fig. 1). At the patient level, high risk patients will receive the PRiVENT intervention which includes expert advice from the weaning centres through newly developed weaning boards and weaning consults. Transfer to the weaning centre will also be possible. The study will end for the patient with discharge from the ICU or from the weaning centre. Other intervention measures include training of health care staff through e-learning opportunities and continuous bedside education. Public relations work is intended to raise awareness of the topic at all levels of society. The intervention was specified according to TIDieR (Template for Intervention Description and Replication), see online Supplement.

Figure 1 shows the expected working mechanisms of the PRiVENT intervention.

### Outcomes

The primary outcome is defined as the successful weaning from IMV of high-risk patients before discharge from the ICU or from the weaning centre.

Secondary outcomes are:

- Time from intubation to successful weaning during hospitalisation in hours on the ventilator.
- Successful weaning for at least 48 consecutive hours in the intervention group, regardless of patient ventilatory status at discharge.
- Mortality during the in-patient stay.
- Re-hospitalisation for the same indication within 30 days of discharge.
- Use of emergency services within 30 days and within 3 months of discharge.
- No need for IMV 3 months after discharge.
- Utilisation of rehabilitation services within 3 months of discharge.
- Re-hospitalisation with IMV within 6 months of discharge.
- Mortality at 6 and 12 months.

Patients who either die before successful weaning, require continued IMV at hospital discharge, or cannot be discharged within 6 months of starting IMV or by 30th June, 2023, are considered weaning failures. June 30th, 2023, is the last date for which comparison data are available and thus represents a pragmatic threshold to ensure comparability of the intervention and comparison groups.

### Intervention elements

#### Prognostic model

Using predictive modelling methods, a prognostic model for prolonged IMV and weaning failure was developed by aQua and Thoraxklinik Heidelberg in several steps. First, a systematic literature review of risk factors associated with prolonged IMV and weaning failure was conducted [30]. Second, based on health care claims data from the AOK-BW, characteristics of in-patients were exploratively identified that had shown an empirical association with an increased risk of subsequent long-term IMV in bivariate analyses. The identified potential predictors were then tested for their predictive effect in multivariable logistic regression models. The goal of this modelling procedure was to identify patients eligible for the PRiVENT intervention. During the intervention patients will be screened and if they are eligible for the study their risk for prolonged IMV and weaning failure will be assessed by the prognostic model after inclusion.

#### Weaning board

After patients are classified into the high-risk group by the prognostic model, the baseline data form is completed in an electronic case report form (eCRF) and submitted to the weaning centre by the ICU. From the weaning centre side, the weaning board consists of at least one respiratory physician with expertise in weaning and one respiratory therapist. Participation of the ICU and other specialists takes place as needed. The weaning board should take place twice a week, at least once a week in the weaning centre. Colleagues from the cooperating ICU can take part in person or via telecommunication. The board’s assessment and recommendations will be communicated to the cooperating clinic pseudonymised via electronic fax or email. The goal is an individualised treatment recommendation for the ICU after an interdisciplinary discussion of the current case. Re-presentations can be made using the progress form. More detailed information can be found in the online Supplement.

#### Weaning consult

The weaning consult is defined as an interdisciplinary case discussion by the PRiVENT weaning centre team with the PRiVENT ICU practitioners. Preferably, the weaning consult takes place at bedside. The first weaning consult of a patient should be performed by the physician and respiratory therapist of the PRiVENT weaning centre within one week of inclusion. For further consults it is sufficient if one of the two is on site. In collaboration with the PRiVENT ICU staff, measures are discussed and a follow-up appointment is made as necessary.

#### Discharge management

As part of PRiVENT’s discharge management, all high-risk patients are followed-up with a structured interview five to eight weeks after discharge by telephone by a member of the study office at the Thorax Clinic Heidelberg. There is scientific evidence that monitoring patients after hospitalisation can allow early detection of complications and avoid re-hospitalisation [31].

#### Interprofessional quality circles, benchmarking & feedback reports

Quality circles are performed by each weaning centre and are interactive discussions of smaller groups, ideally no more than eight to 15 participants. A trained moderator (respiratory physician or respiratory therapist) leads the topic-centred discussion among participating colleagues. Positive and negative aspects of current processes are identified in order to initiate improvements. New findings are recorded and concrete measures are developed to be implemented for quality improvement. The latter are reviewed as part of the final evaluation. In PRiVENT quality circles take place every six month in person or online and take about one to two hours. Participants include staff from the cooperating ICU.

#### E-learning & training

Seven e-learning modules on weaning and corresponding case studies have been developed. These are made available online to the health care professional (physicians and nurses) participating in the study programme via a personal login code. The number and distribution of users in regard to the cooperating clinic are documented and evaluated. In addition, each weaning centre provides additional training once during the intervention phase.

#### Public relations and patients’ involvement

PRiVENT’s public relations work reaches out to relatives of ventilated patients, high-risk patients, medical professionals, and the general public. PRiVENT aims to educate the general public about the options and positive effects of weaning to minimise the risk of long-term IMV. A project website has been established with blog posts and a podcast series to provide reliable and trustworthy information. The information is published on high-reach platforms, such as Facebook, Instagram, Spotify and YouTube. Additionally, there is a cooperation with “Gesundheitstreffpunkt Mannheim”, a charitable organization with the objective to improve patients’ information and support groups.

### Recruitment of weaning centres and intensive care units

Only DGP (German Respiratory Society)-certified weaning units in BW are eligible to participate as weaning centres. All ICUs in BW that are not recruited as weaning centres can participate as ICUs.

### Recruitment of patients

#### Intervention study experimental group

The experimental group of the intervention study will consist of patients recruited from PRiVENT ICUs. All patients in participating centres on IMV between 96 h and 11 days will be screened for eligibility. This timeframe has been chosen pragmatically to ensure timely assignment of eligible patients to the intervention. Patients who fulfil the eligibility criteria and have given informed consent to participate in the study can be included. As invasively ventilated patients are usually not capable of giving consent, their legal guardian will be informed instead. After undergoing the prognostic model that classifies patients into high- and low-risk groups for long-term IMV, high-risk and all COVID-19 patients will be assigned to the intervention.

Inclusion criteria:

- ≥ 96 h of invasive ventilation
- no more than 7 days have passed since the patient completed their 96th hour of invasive ventilation
- ≥ 30 years old
- ≥ 1 comorbidity and/or acute Covid-19 pneumonia
- not suffering from any neuromuscular disease without weaning potential

No further exclusion criteria.

The time schedule of the patients’ journey can be found in the Supplement.

#### Comparison group of the intervention study

The comparison group will be matched from AOK-BW health care claims data provided by hospitals in Baden-Württemberg. Because of the necessary processing in AOK-BW the data will be available from late 2019 to late 2023. Only patients ventilated for at least 96 h will be included in the comparison group. Patient data will then be reviewed for the other eligibility criteria and the risk of long-term IMV will be assessed by the prognostic model. Patients admitted to the hospital with acute COVID-19 pneumonia are automatically classified as high-risk. The group of patients used for the primary assessment of the intervention effect will be additionally limited to patients who do not die within 11 days of starting ventilation. This ensures comparability with the intervention group and corresponds to the screening period in the intervention group.

### Data collection

Data collection in the patient trial group will be conducted via an eCRF by designated ICU staff, weaning centres and the study centre using Redcap (Research Electronic Data Capture). The data of the comparison group is generated from AOK-BW health care claims data, delivered to the aQua Institute (Institute for Applied Quality Promotion and Research in Health Care) and processed there and analysed in the IMBI (Institute of Medical Biometry and Informatics). Data from the e-learning will be evaluated via the Moodle platform, the central learning platform of the University of Heidelberg. Publicity is recorded on the basis of visits/clicks, which is provided by the respective platforms. More detailed information can be found in the Supplement.

### Sample size

For the purpose of power estimation, we assumed that 1,495 high-risk patients per group would be recruited. Further, we assumed patients are recruited in 40 cooperating ICU with an average cluster size of 25 and an intra-class correlation coefficient of 0.1, yielding a design effect coefficient of 3.4 [32]. Incorporating this into our power calculation would give us an effective sample size of 439 in both groups. Presuming a drop-out rate of 10.6%, we would reach an effective sample size of 393 in both groups. Assuming a rate of 20% weaning failures in the intervention and 30% in the comparison group, a chi-squared test with a significance level of 5% would have a power of 90% to detect a difference between the treatment and the comparison group. The actual primary analysis will be based on a mixed logistic regression model adjusted for various confounders including correlations within clusters, which can be expected to have better operating characteristics than a chi-squared test. This power analysis was conducted using PASS version 16.0.3.

### Analysis

#### Interim analysis

There will be an interim analysis after 12 months of intervention and a final analysis at the end of the entire intervention period. The aim of the interim analysis is to assess the recruitment effort of the centres and the influence of COVID-19 on the outcomes. The results are accessible to the PRiVENT study group.

#### Final analysis

The primary outcome, successful weaning before discharge, will be evaluated using a mixed logistic regression model including treatment group, age, gender, acute COVID-19 pneumonia, pre-existing medical conditions via the Charlson Comorbidity Index (continuously) [33], number of ventilation cases of the centre, dependency on ventilatory assistance for at least 3 month within 1 year before admission (with tracheostomy vs without tracheostomy vs none), tracheostomy within 96 h of intubation, extracorporeal support (yes/no), and centre as a random intercept. The analysis population for the primary endpoint will be restricted in order that a possible bias due to the selection effect of the screening window will be minimized. If necessary, missing values will be imputed via a multiple imputation approach. For patients included into the analysis population, intercurrent events (such as death) which would prevent patients’ successful weaning will be counted as weaning failures. A number of sensitivity analysis will be conducted to investigate the impact of the imputation strategy, the 7-day inclusion window, and the choice of covariates.

The analysis of secondary outcomes will include the calculation of appropriate summary measures of the respective empirical distributions (mean, standard deviation, median, interquartile range, minimum and maximum for continuous variables, and absolute and relative frequencies for categorical variables), as well as descriptive *p*-values. In addition, similar mixed models as described for the primary outcome will be used, logistic models for categorical outcomes and linear models for continuous outcomes. Time to event endpoints will be analysed using methods from survival analysis, including Kaplan–Meier estimates, log-rank tests and shared frailty models similar to the primary analysis model. As appropriate, graphical methods will be used to visualise the results. Missing data in secondary outcomes will not be imputed.

In an explorative analysis, clinical data collected within the trial will be used in to identify potentially relevant factors which could help to supplement the developed prognosis model. To this end, an elastic-net approach, as well as decision-tree based models will be used.

A detailed statistical analysis plan will be set up prior to the final analysis. R version 4.0.2 or higher (www.R-project.org) will be used to carry out the analyses.

### Data protection

Data protection is guaranteed in accordance with the European Data Protection Regulation, the Baden-Württemberg State Data Protection Act, and the Federal Data Protection Act. Furthermore, the data protection concept has been reviewed and accepted by the data protection officer of the Heidelberg University Hospital.

## Discussion

### Strenghts

- The study will draw attention to weaning and all of its facets
- The study allows to share expertise and improve patient care in clinics without weaning expertise
- The study will improve the collaboration between the intervention team, consisting of specialised weaning physicians and respiratory therapists, the weaning centres and intensive care personal and thus strengthen the role of specialised personal

### Limitations

- Intervention procedures may be applied heterogeneously in this field study
- The study is not blinded and not randomised

## Supplementary Information

**Additional file 1.** TIDieR Checklist.

## Data Availability

Results are planned to be published in open access journals and reports will be available at the funding institution. The datasets used and analysed during the current study are available from the corresponding author on reasonable request.

## Abbreviations

AOK-BW: Allgemeine Ortskrankenkasse Baden-Württemberg
eCRF: Electronic case report form
ICU: Intensive care unit
IMV: Invasive mechanical ventilation
PRiVENT: Prevention of invasive ventilation
