# Supplementary material for: Prevention of invasive ventilation (PRiVENT)—a prospective, mixed-methods interventional, multicentre study with a parallel comparison group: study protocol
Source: BMC Health Serv Res. 2023 Mar 30;23:305. doi: 10.1186/s12913-023-09283-0 (PMC10061400; doi:10.1186/s12913-023-09283-0)
Supplement: Supplementary file 1 — Additional file 1. TIDieR Checklist. [file 12913_2023_9283_MOESM1_ESM.docx]

1. **TIDieR Checklist**

**
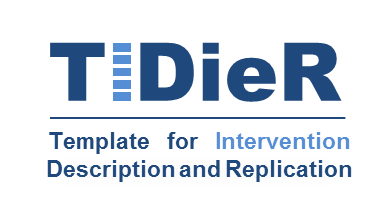
The TIDieR (Template for Intervention Description and Replication) Checklist*:**

Information to include when describing an intervention and the location of the information

| **Item number**  **1** | **Item** | **Where located **** | |
| --- | --- | --- | --- |
|  | Prognostic model | Primary paper  (page or appendix  number) | Other ^†^ (details) |
|  | **BRIEF NAME** |  |  |
| **1.** | Provide the name or a phrase that describes the intervention. | 7, 161 | ______________ |
|  | **WHY** |  |  |
| **2.** | Describe any rationale, theory, or goal of the elements essential to the intervention. | 7, 168 | _____________ |
|  | **WHAT** |  |  |
| **3.** | Materials: Describe any physical or informational materials used in the intervention, including those provided to participants or used in intervention delivery or in training of intervention providers. Provide information on where the materials can be accessed (e.g. online appendix, URL). | 7, 163 | _____________ |
| **4.** | Procedures: Describe each of the procedures, activities, and/or processes used in the intervention, including any enabling or support activities. | 7, 163 | _____________ |
|  | **WHO PROVIDED** |  |  |
| **5.** | For each category of intervention provider (e.g. psychologist, nursing assistant), describe their expertise, background and any specific training given. | 7, 163 | _____________ |
|  | **HOW** |  |  |
| **6.** | Describe the modes of delivery (e.g. face-to-face or by some other mechanism, such as internet or telephone) of the intervention and whether it was provided individually or in a group. | 7, 163 | _____________ |
|  | **WHERE** |  |  |
| **7.** | Describe the type(s) of location(s) where the intervention occurred, including any necessary infrastructure or relevant features. | 7, 163 | _____________ |
|  | **WHEN and HOW MUCH** |  |  |
| **8.** | Describe the number of times the intervention was delivered and over what period of time including the number of sessions, their schedule, and their duration, intensity or dose. | N/A | _____________ |
|  | **TAILORING** |  |  |
| **9.** | If the intervention was planned to be personalised, titrated or adapted, then describe what, why, when, and how. | N/A | _____________ |
|  | **MODIFICATIONS** |  |  |
| **10.^ǂ^** | If the intervention was modified during the course of the study, describe the changes (what, why, when, and how). | N/A | _____________ |
|  | **HOW WELL** |  |  |
| **11.** | Planned: If intervention adherence or fidelity was assessed, describe how and by whom, and if any strategies were used to maintain or improve fidelity, describe them. | N/A | _____________ |
| **12.^ǂ^** | Actual: If intervention adherence or fidelity was assessed, describe the extent to which the intervention was delivered as planned. | N/A | _____________ |

| **Item number**  **2** | **Item** | **Where located **** | |
| --- | --- | --- | --- |
|  | Weaning Board | Primary paper  (page or appendix  number) | Other ^†^ (details) |
|  | **BRIEF NAME** |  |  |
| **1.** | Provide the name or a phrase that describes the intervention. | 7, 172 | ______________ |
|  | **WHY** |  |  |
| **2.** | Describe any rationale, theory, or goal of the elements essential to the intervention. | 7, 180 | _____________ |
|  | **WHAT** |  |  |
| **3.** | Materials: Describe any physical or informational materials used in the intervention, including those provided to participants or used in intervention delivery or in training of intervention providers. Provide information on where the materials can be accessed (e.g. online appendix, URL). | N/A | _____________ |
| **4.** | Procedures: Describe each of the procedures, activities, and/or processes used in the intervention, including any enabling or support activities. | 7, 175 | _____________ |
|  | **WHO PROVIDED** |  |  |
| **5.** | For each category of intervention provider (e.g. psychologist, nursing assistant), describe their expertise, background and any specific training given. | 7, 175 | _____________ |
|  | **HOW** |  |  |
| **6.** | Describe the modes of delivery (e.g. face-to-face or by some other mechanism, such as internet or telephone) of the intervention and whether it was provided individually or in a group. | 7, 177 | _____________ |
|  | **WHERE** |  |  |
| **7.** | Describe the type(s) of location(s) where the intervention occurred, including any necessary infrastructure or relevant features. | 7, 177 | _____________ |
|  | **WHEN and HOW MUCH** |  |  |
| **8.** | Describe the number of times the intervention was delivered and over what period of time including the number of sessions, their schedule, and their duration, intensity or dose. | 7, 177 | Online Supplement 3 |
|  | **TAILORING** |  |  |
| **9.** | If the intervention was planned to be personalised, titrated or adapted, then describe what, why, when, and how. | N/A | _____________ |
|  | **MODIFICATIONS** |  |  |
| **10.^ǂ^** | If the intervention was modified during the course of the study, describe the changes (what, why, when, and how). | N/A | _____________ |
|  | **HOW WELL** |  |  |
| **11.** | Planned: If intervention adherence or fidelity was assessed, describe how and by whom, and if any strategies were used to maintain or improve fidelity, describe them. | N/A | _____________ |
| **12.^ǂ^** | Actual: If intervention adherence or fidelity was assessed, describe the extent to which the intervention was delivered as planned. | N/A | _____________ |

| **Item number**  **3** | **Item** | **Where located **** | |
| --- | --- | --- | --- |
|  | Weaning Consult | Primary paper  (page or appendix  number) | Other ^†^ (details) |
|  | **BRIEF NAME** |  |  |
| **1.** | Provide the name or a phrase that describes the intervention. | 8, 184 | ______________ |
|  | **WHY** |  |  |
| **2.** | Describe any rationale, theory, or goal of the elements essential to the intervention. | 8, 185 | _____________ |
|  | **WHAT** |  |  |
| **3.** | Materials: Describe any physical or informational materials used in the intervention, including those provided to participants or used in intervention delivery or in training of intervention providers. Provide information on where the materials can be accessed (e.g. online appendix, URL). | N/A | _____________ |
| **4.** | Procedures: Describe each of the procedures, activities, and/or processes used in the intervention, including any enabling or support activities. | 8, 187 | _____________ |
|  | **WHO PROVIDED** |  |  |
| **5.** | For each category of intervention provider (e.g. psychologist, nursing assistant), describe their expertise, background and any specific training given. | 8, 187 | _____________ |
|  | **HOW** |  |  |
| **6.** | Describe the modes of delivery (e.g. face-to-face or by some other mechanism, such as internet or telephone) of the intervention and whether it was provided individually or in a group. | 8, 186 | _____________ |
|  | **WHERE** |  |  |
| **7.** | Describe the type(s) of location(s) where the intervention occurred, including any necessary infrastructure or relevant features. | 8, 186 | _____________ |
|  | **WHEN and HOW MUCH** |  |  |
| **8.** | Describe the number of times the intervention was delivered and over what period of time including the number of sessions, their schedule, and their duration, intensity or dose. | 8, 188 |  |
|  | **TAILORING** |  |  |
| **9.** | If the intervention was planned to be personalised, titrated or adapted, then describe what, why, when, and how. | N/A | _____________ |
|  | **MODIFICATIONS** |  |  |
| **10.^ǂ^** | If the intervention was modified during the course of the study, describe the changes (what, why, when, and how). | N/A | _____________ |
|  | **HOW WELL** |  |  |
| **11.** | Planned: If intervention adherence or fidelity was assessed, describe how and by whom, and if any strategies were used to maintain or improve fidelity, describe them. | N/A | _____________ |
| **12.^ǂ^** | Actual: If intervention adherence or fidelity was assessed, describe the extent to which the intervention was delivered as planned. | N/A | _____________ |
| **Item number**  **4** | **Item** | **Where located **** | |
|  | Discharge management | Primary paper  (page or appendix  number) | Other ^†^ (details) |
|  | **BRIEF NAME** |  |  |
| **1.** | Provide the name or a phrase that describes the intervention. | 8, 191 | ______________ |
|  | **WHY** |  |  |
| **2.** | Describe any rationale, theory, or goal of the elements essential to the intervention. | 8, 194 | _____________ |
|  | **WHAT** |  |  |
| **3.** | Materials: Describe any physical or informational materials used in the intervention, including those provided to participants or used in intervention delivery or in training of intervention providers. Provide information on where the materials can be accessed (e.g. online appendix, URL). | N/A | _____________ |
| **4.** | Procedures: Describe each of the procedures, activities, and/or processes used in the intervention, including any enabling or support activities. | 8, 192 | _____________ |
|  | **WHO PROVIDED** |  |  |
| **5.** | For each category of intervention provider (e.g. psychologist, nursing assistant), describe their expertise, background and any specific training given. | 8, 193 | _____________ |
|  | **HOW** |  |  |
| **6.** | Describe the modes of delivery (e.g. face-to-face or by some other mechanism, such as internet or telephone) of the intervention and whether it was provided individually or in a group. | 8, 193 | _____________ |
|  | **WHERE** |  |  |
| **7.** | Describe the type(s) of location(s) where the intervention occurred, including any necessary infrastructure or relevant features. | 8, 193 | _____________ |
|  | **WHEN and HOW MUCH** |  |  |
| **8.** | Describe the number of times the intervention was delivered and over what period of time including the number of sessions, their schedule, and their duration, intensity or dose. | 8, 193 |  |
|  | **TAILORING** |  |  |
| **9.** | If the intervention was planned to be personalised, titrated or adapted, then describe what, why, when, and how. | N/A | _____________ |
|  | **MODIFICATIONS** |  |  |
| **10.^ǂ^** | If the intervention was modified during the course of the study, describe the changes (what, why, when, and how). | N/A | _____________ |
|  | **HOW WELL** |  |  |
| **11.** | Planned: If intervention adherence or fidelity was assessed, describe how and by whom, and if any strategies were used to maintain or improve fidelity, describe them. | N/A | _____________ |
| **12.^ǂ^** | Actual: If intervention adherence or fidelity was assessed, describe the extent to which the intervention was delivered as planned. | N/A | _____________ |
| **Item number**  **5** | **Item** | **Where located **** | |
|  | Quality circle | Primary paper  (page or appendix  number) | Other ^†^ (details) |
|  | **BRIEF NAME** |  |  |
| **1.** | Provide the name or a phrase that describes the intervention. | 8, 196 | ______________ |
|  | **WHY** |  |  |
| **2.** | Describe any rationale, theory, or goal of the elements essential to the intervention. | 8, 201 | _____________ |
|  | **WHAT** |  |  |
| **3.** | Materials: Describe any physical or informational materials used in the intervention, including those provided to participants or used in intervention delivery or in training of intervention providers. Provide information on where the materials can be accessed (e.g. online appendix, URL). | N/A | _____________ |
| **4.** | Procedures: Describe each of the procedures, activities, and/or processes used in the intervention, including any enabling or support activities. | 8, 198 | _____________ |
|  | **WHO PROVIDED** |  |  |
| **5.** | For each category of intervention provider (e.g. psychologist, nursing assistant), describe their expertise, background and any specific training given. | 8, 198 | _____________ |
|  | **HOW** |  |  |
| **6.** | Describe the modes of delivery (e.g. face-to-face or by some other mechanism, such as internet or telephone) of the intervention and whether it was provided individually or in a group. | 8, 202 | _____________ |
|  | **WHERE** |  |  |
| **7.** | Describe the type(s) of location(s) where the intervention occurred, including any necessary infrastructure or relevant features. | 8, 202 | _____________ |
|  | **WHEN and HOW MUCH** |  |  |
| **8.** | Describe the number of times the intervention was delivered and over what period of time including the number of sessions, their schedule, and their duration, intensity or dose. | 8, 202 |  |
|  | **TAILORING** |  |  |
| **9.** | If the intervention was planned to be personalised, titrated or adapted, then describe what, why, when, and how. | N/A | _____________ |
|  | **MODIFICATIONS** |  |  |
| **10.^ǂ^** | If the intervention was modified during the course of the study, describe the changes (what, why, when, and how). | N/A | _____________ |
|  | **HOW WELL** |  |  |
| **11.** | Planned: If intervention adherence or fidelity was assessed, describe how and by whom, and if any strategies were used to maintain or improve fidelity, describe them. | N/A | _____________ |
| **12.^ǂ^** | Actual: If intervention adherence or fidelity was assessed, describe the extent to which the intervention was delivered as planned. | N/A | _____________ |

** **Authors** - use N/A if an item is not applicable for the intervention being described. **Reviewers** – use ‘?’ if information about the element is not reported/not sufficiently reported.

† If the information is not provided in the primary paper, give details of where this information is available. This may include locations such as a published protocol or other published papers (provide citation details) or a website (provide the URL).

ǂ If completing the TIDieR checklist for a protocol, these items are not relevant to the protocol and cannot be described until the study is complete.

* We strongly recommend using this checklist in conjunction with the TIDieR guide (see *BMJ* 2014;348:g1687) which contains an explanation and elaboration for each item.

* The focus of TIDieR is on reporting details of the intervention elements (and where relevant, comparison elements) of a study. Other elements and methodological features of studies are covered by other reporting statements and checklists and have not been duplicated as part of the TIDieR checklist. When a **randomised trial** is being reported, the TIDieR checklist should be used in conjunction with the CONSORT statement (see [www.consort-statement.org](http://www.consort-statement.org)) as an extension of **Item 5 of the CONSORT 2010 Statement.** When a **clinical trial** **protocol** is being reported, the TIDieR checklist should be used in conjunction with the SPIRIT statement as an extension of **Item 11 of the SPIRIT 2013**

**2. Figure 2: Patient journey**


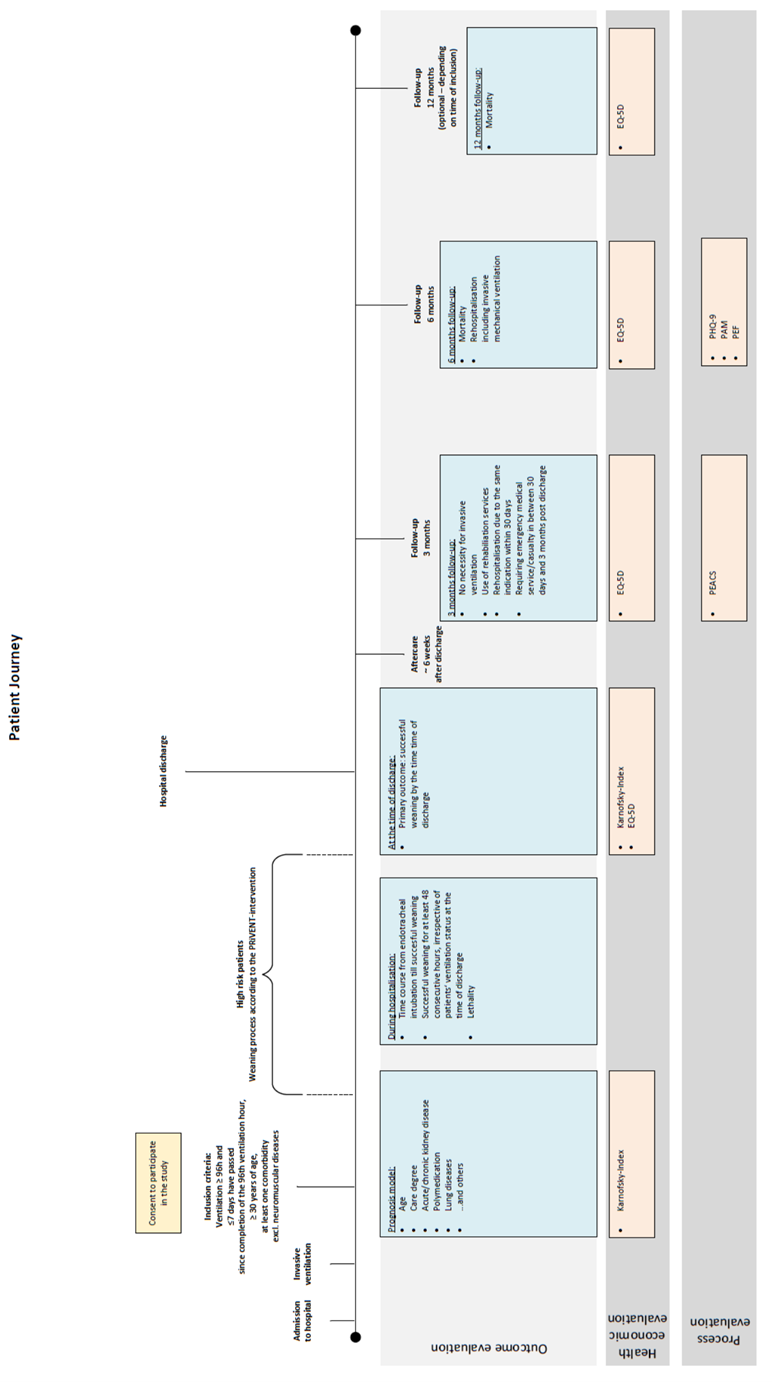


**3. More detailed information on the Weaning Board**

The Weaning Board is meant to be an interdisciplinary case conference, held on a regular basis, to optimise weaning for patients depending on invasive ventilation. Ideas to realise such meetings already exist at the St. Marien-Hospital in Cologne (Schlesinger et al 2018). Patient cases have been discussed in retrospect. Still, Schlesinger et al. report overall improvement of patient outcome, shortened length of stay in hospital, better training of those professions involved and reduction in employee turnover.

In PRiVENT, the Weaning Board will serve as a platform to exchange information and discuss patient cases in order to evaluate patients’ weaning potential and to give individualised treatment advice to the cooperating clinic (external intensive care unit). This approach aims to advance the overall weaning process outside specialised weaning centres by focussing on patients’ inhaled therapy regarding their lung function and respiratory pump as well as drug management.

The Weaning Board, at minimum, is set up by the respiratory physician with expertise in weaning as well as the respiratory therapist. Other professions, e.g. psychosocial services, intensive care personnel or physiotherapists may be consulted by the board. If necessary, the Weaning Board might also invite their colleagues, the responsible doctor or nurse, from the cooperating clinic to join the meeting via videoconference.

All patients enrolled in the study will be introduced to the interprofessional board. Patients may be discussed several times during their in-patient stay till treatment is completed. Therefore, the cooperating clinic (external intensive care unit) will need to register each newly enrolled patient with the Weaning Board. The Weaning Board will hold a meeting twice a week in the premises of the weaning centre if a patient is registered either for initial consultation or follow-up. Weekdays may differ in the participating weaning centres.

The patient’s history will be pseudonymised and sent to the weaning centre via electronic fax or email right after inclusion and application of the prognosis model. In case the Weaning Board does not meet at the time of inclusion, a follow-up protocol will be filled in and send to the weaning centre right before the next Weaning Board holds its meeting.

In detail, the board members of the supervising weaning centre will consider patients’ state of consciousness, whether or not patients are able to breath spontaneously, whether there are expanding spontaneous breathing phases, and finally deliberating if and when to extubate or decannulate the patients discussed. Hereafter, they will review the treatment of adjunctive problems such as delirium, cardiac dysfunction, infections and comorbid organ failure.

The board’s assessment and recommendations concerning the next therapeutic steps will be documented and communicated to the cooperating clinic. Unless the Weaning Board will recommend transferring the patient to the weaning centre, the cooperating clinic will receive the board’s recommendations together with an appointment for re-assessment in writing. In case a patient needs to be transferred to the responsible weaning centre, the patient’s individual treatment plan will still be discussed by the interprofessional Weaning Board.

The need for intervention will be assessed for each patient and re-evaluated by the Weaning Board, thereby considering the cases’ complexity and demand for advice. In severe cases, board members will either decide to visit on-site or reschedule registration with the Weaning Board. In cases with less need for advice, recommendation will be given in writing.

In order to meet the obstacles in the complex weaning process, all aspects must be addressed and appraised by the interprofessional team. For this purpose, three different forms, an anamnesis questionnaire, a follow-up protocol as well as an assessment form, will be developed during the pilot phase. These forms will be integrated in the eCRF. Printouts of these will allow submission of patients’ pseudonymised data via electronic fax or email and serve the Weaning Board’s consultation. Data will also be used for evaluation purposes in a descriptive manner. The analysis of this data will allow better characterisation of the intervention group and might further contribute to adjust current management plans. The anamnesis questionnaire will be filled in and forwarded by the cooperating clinic each time a patient is registered with the Weaning Board. For re-assessment, the follow-up protocol will be submitted by the cooperating clinic on the day of the patient’s next appointment with the Weaning Board. If the consultation needs to take place on-site, the Weaning Board will contact the cooperating clinic by phone to schedule the Weaning Consultation.

## **4. More detailed information on the Data collection and data protection concept**

Access to personal data of patients will be granted to those who are involved in the treatment of the patient participating in PRiVENT. In addition, to the treatment team on-site, this will also apply to experts who are consulted from outside, i.e., the corresponding weaning centres.

Data collected for evaluation purposes during the pilot and intervention study will be pseudonymised and documented using electronic Case Report Forms (eCRF) set up in REDCap (Research Electronic Data Capture), a secure web application for building and managing online surveys and databases. Data will be encrypted using the patient’s pseudonym. The latter will be a combination of numbers defined by the study central office. It is made up by the ID given to the weaning-centre, the ID given to the participating intensive care unit as well as the patient’s screening number which will be generated by the eCRF when recording the screening process.

The pseudonymisation key will therefore be managed by the respective intensive care units and weaning centres who conduct the intervention. Data collection will comprise patients’ personal details (age, sex), care degree, information on previous illnesses, current diseases, haemodialysis, course of ventilation including details on tracheotomy, laboratory and ventilation parameters, whether and by which profession the intervention elements Weaning Board or Weaning Consultation were conducted, the outcome of the intervention and whether there were any problems around the time of discharge. Clinical data will be recorded on the ward or at the patient's bed using a tablet PC or workstation. Weaning centres will regularly document data concerning the conduction of the Weaning Board and Weaning Consultation as described earlier, and in case patients will be transferred they will also record the beforementioned clinical data and patients’ intervention outcome. Aftercare will be documented by the study centre at the Thorax Clinic Heidelberg. All recorded data collected in the participating clinics and weaning centres will be transmitted to a server at the University Hospital Heidelberg that is protected from external access using hypertext transfer protocol secure (https) as well as secure Virtual Private Network (VPN) protocols.

Data collection via the eCRF will be processed in the Department of General Practice and Health Services Research and made available at regular intervals to the aQua-Institute for the preparation of feedback reports via Secure File Transfer Protocol (SFTP). Since the feedback reports are meant to address each participating clinic individually and will be printed by the aQua-Institute, shortly before the print, the hospitals’ ID number will be decoded while patient data remain pseudonymised.

Pseudonymised healthcare claims data will be provided by the AOK-BW to create the prognosis model and to evaluate the control group. For this purpose, data will be transmitted to the aQua-Institute via SFTP. During the first year of the study, data will be available in December 2020, and in subsequent years by the end of the third quarter at the latest. The aQua-Institute will process the beforementioned data together with the data of the intervention group and provide the again pseudonymised data to the Institute of Medical Biometry and Informatics via SFTP for evaluation purposes, at the time of the interim and final analyses.

Data will be stored and retained at the study central office for a period of 10 years. As soon as the study’s purposes are fulfilled, all personal data will be anonymised according to article 89 sentence 1 of the General Data Protection Act. After the retention period has expired, data will be destructed.

Only pseudonymised data will be used for the evaluation of PRiVENT. Participants who will give consent to take part in the study or whose legal proxy has given consent on their behalf, also agree to data processing according to Art. 6 para. 1 lit. a of the General Data Protection Regulation as well as provision of pseudonymised healthcare claims data. Because consent has already been granted according to Art. 75 para. 1 sentence 2 SGB X and because of the unreasonableness, a new declaration of consent will not be obtained for the control group.

The provisions of the Baden-Württemberg State Data Protection Act and the Federal Data Protection Act will be observed. All responsibilities regarding data management and analysis as well as access to the data is laid down in a separate data protection concept, which is part of the contractual agreement between the consortium partners and will be approved by the data protection officer at the University Hospital Heidelberg.

**5. Weaning centres and associated ICUs (recruiting ongoing)**

1. Weaning centre Thoraxklinik Heidelberg – ICUs

- University Hospital Heidelberg Gastroenterology
- University Hospital Heidelberg Surgery
- University Hospital Heidelberg Cardiology
- University Hospital Heidelberg Orthopedics (initiation planned)
- St. Josefskrankenhaus Heidelberg
- Siloah Klinikum Pforzheim
- GRN Kliniken Schwetzingen
- University Hospital Mannheim Internal Medicine
- University Hospital Mannheim Anaesthetics
- Städtisches Krankenhaus Karlsruhe Internal Medicine
- Helios Klinikum Pforzheim
- SRH Kurpfalzkrankenhaus Heidelberg

2. Weaning centre SLK Lungenklinik Löwenstein– ICUs

- Caritas Krankenhaus Bad Mergentheim
- Ostalb Kliniken Ellwangen
- Ostalb Kliniken Aalen
- SLK Klinikum am Plattenwald
- Klinikum Crailsheim
- Klinikum Heidenheim
- Hohenloher Krankenhaus Öhringen
- SLK Klinikum am Gesundbrunnen
- SRH Klinikum Karlsbad
- Diakonie-Klinikum Schwäbisch Hall Internal Medicine (initiation planned)
- Diakonie-Klinikum Schwäbisch Hall Anaesthetics (initiation planned)

3. Weaning centre Robert Bosch Krankenhaus Klinik Schillerhöhe– ICUs

- Robert-Bosch Krankenhaus Cardiology
- Robert-Bosch Krankenhaus Anaesthetics
- Filderklinik Filderstadt-Bonlanden
- University Hospital Tübingen Internal Medicine
- University Hospital Tübingen Anaesthetics
- Krankenhäuser Landkreis Freudenstadt
- Zollernalb Klinikum Albstadt/Balingen
- Klinikum Tuttlingen
- Diakoniekrankenhaus Stuttgart
- Kreiskliniken Reutlingen

4. Weaning centre Waldburg Zeil Kliniken Fachklinik Wangen– ICUs

- Oberschwabenklinik, St. Elisabethen Klinikum Ravensburg
- Hegau-Bodensee Klinikum Singen
- RKU Universitäts-und Rehakliniken Ulm
- Krankenhaus Alb-Donau-Kreis Standort Blaubeuren
- Krankenhaus Alb-Donau-Kreis Standort Ehingen
- Oberschwabenklinik Westallgäu-Klinikum Wangen
- MCB (Medizin Campus Bodensee) Klinikum Friedrichshafen
